# The legacy of the COVID-19 pandemics for thyroid cancer patients: towards the application of clinical practice recommendations

**DOI:** 10.1007/s12020-022-03132-6

**Published:** 2022-07-20

**Authors:** Giorgio Grani, Laura Ciotti, Valeria Del Gatto, Teresa Montesano, Marco Biffoni, Laura Giacomelli, Marialuisa Sponziello, Valeria Pecce, Antonella Verrienti, Sebastiano Filetti, Cosimo Durante

**Affiliations:** 1 Department of Translational and Precision Medicine, Sapienza University of Rome, Viale del Policlinico 155, 00161 Rome, Italy; 2 Department of Surgical Sciences, Sapienza University of Rome, Viale del Policlinico 155, 00161 Rome, Italy; 3 School of Health, UNITELMA Sapienza University of Rome, Piazza Sassari, 4, 00161 Rome, Italy

The outbreak of COVID-19 pandemic has acted as a significant stress test for healthcare systems worldwide, due to the need for hospitalization of an increasing number of infected patients. The shift of massive resources to the acute needs of the pandemic led to an upheaval of the usual diagnostic and therapeutic pathways of chronic diseases, including thyroid cancer disease. The *motto* was to reduce crowding at clinics and to maintain essential health services. However, thyroid cancer clinical practice recommendations already encouraged physicians to reduce “low-value” care: in particular, to avoid screening of general population, to reduce the number of unnecessary biopsies, and to adopt a conservative approach to indeterminate thyroid nodules and low-risk thyroid cancers [1].

## Recommendations from oncology scientific societies

In 2020, the European Society for Medical Oncology published an Interdisciplinary Expert Consensus on the management of cancer patients. The experts recommended that cancer care intensity be adapted to the pandemic scenario, to local R0 index and to health facilities and resources [2].

This was soon after followed by specific guidance documents on single cancer types, palliative, and supportive care. As for thyroid cancer treatment prioritization, newly diagnosed patients with clinical T4, bulky lymph node involvement, or aggressive histotypes (such as medullary, tall-cell variant of papillary thyroid cancer, poorly differentiated or anaplastic thyroid cancer) were considered high priority for primary surgery; clinical T2 and T3 with no extensive lymph node involvement as medium priority; and clinical T1 or indeterminate cytology nodules were classified as low priority [2]. It is worthy of note that according to recent data from an Italian country-wide prospective observational study, high-priority cases account for about 8% of all thyroid cancer diagnoses [3]. Furthermore, a UK modeling study estimated that a 3-month delay in diagnosis has an effect of less than 3% on 10-year thyroid cancer mortality risk, for all age groups [4].

## Participation in cancer screenings

**Fig. 1:**
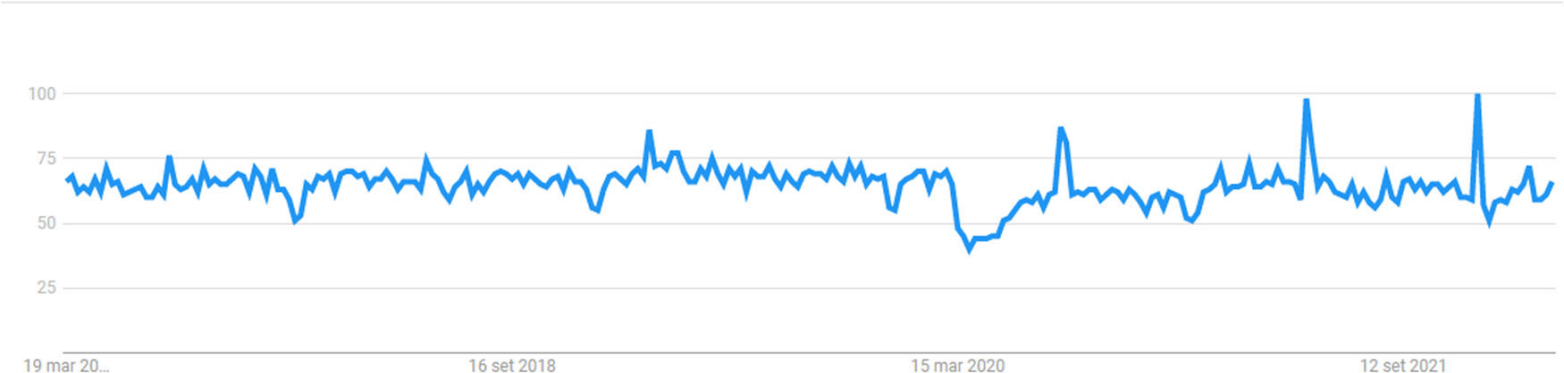
Online interest for papillary thyroid cancer in the last 5 years. Data source: Google Trends (https://www.google.com/trends)

When treatment for low-risk, early-stage cancer is likely to be postponed or avoided altogether, interest in cancer screening programs is expected to diminish. Using the Google Trends tool, Cohen et al. found that there was a significantly reduced public interest in six malignancies used as a benchmark (including thyroid cancer) at the beginning of the COVID-19 pandemic. However, online search volumes returned to pre-pandemic levels in the second half of 2020 [5]. An update of these trends for thyroid cancer is provided in Fig. 1. As a result, in the United States, at the peak of the first wave of the pandemic in April 2020, screenings for breast, colon, prostate, and lung cancers dropped by 85%, 75%, 74%, and 56%, respectively [6].

While this may be worrying for some cancer types, it is not for thyroid cancer. According to the World Health Organization, cancer screenings are justified if the target cancers are frequent; a high proportion of patients is diagnosed in advanced stages; early detection methods are cost-effective and easily accessible for individuals at risk; diagnosis, treatment, follow-up, and quality assurance procedures can be implemented; and the benefits of early detection outweigh the risks (such as complications and negative effects). According to the US Preventive Services Task Force (USPSTF), this is not the case for asymptomatic adults with differentiated thyroid cancer. USPSTF recommends against screening since the potential risks outweigh any potential benefits [7]. Furthermore, thyroid cancers detected by screening are usually small, very low-risk papillary carcinomas, therefore unlikely to affect both survival and quality of life. Also for higher-risk populations (history of exposure to ionizing radiation, or one or more first-degree relatives with a history of thyroid cancer), evidence does not support the implementation of an ultrasound-based screening program [8].

In summary, the decreased interest in thyroid cancer screening is likely to reduce overdiagnosis and overtreatment of indolent thyroid cancer.

## Number of performed biopsies

The COVID-19 pandemic impact on cytopathology practice was investigated both at national and international levels. An international, multicenter study found a persistent reduction in the cytological specimen volume both during the lockdown [9] and the post-lockdown period [10], defined as the first 12 weeks of post-lockdown “representative” practice, between April 4 and October 31, 2020. In particular, thyroid cytopathology specimens decreased by 80.5% during the lockdown, and by 32.8% in the post-lockdown period. Generally speaking, the authors maintain that the continued reduction of processed samples in the post-lockdown period represents a global health care issue, and the relative slight increase in the cytological workload is a promising finding [10].

The thyroid case is, however, peculiar. In fact, scientific societies have released a number of recommendations to better stratify the malignancy risk of thyroid nodules, aiming at an overall reduction of unnecessary fine-needle aspiration biopsies [11]. Interestingly, a strict application of international sonographic stratification systems would lead to a reduction of about 50% of biopsies, similarly to the one observed during the lockdown. Beside the reluctance of patients to go to hospitals or the restrictions on access to clinics, the biopsy de-escalation may well be due to a better risk-stratification performed by radiologists and clinicians, triggered by the new pandemic context.

## Reduction and postponement of thyroid surgeries

In this context, surgical activity for thyroid disease was also reduced [12]. During lockdowns, prioritization systems were applied [13] and thyroidectomy was still performed for clinically-significant thyroid malignancies and uncontrolled hyperthyroidism [14].

A North American survey showed that many endocrine surgeons (37.7%) were reassigned to other duties during the pandemic; most surgeons reported decreased clinical volume (74.6%) [15]. A multicenter Italian study quantified the overall reduction of surgical activity for thyroid disease: 67.8% of surgical units had undergone a reduction in inpatient beds, and half units experienced a reduction in personnel. The decrease in the number of thyroid surgeries was 64.8% during the lockdown period (March–May 2020), 44.7% during the subsequent phase (May–June 2020), and 5.1% in the subsequent summer, all compared to the same periods of the previous year. In the first two phases, the rate of malignancies was significantly higher, as proof of the selection of treated patients. The incidence of post-operative complications was unchanged [16].

In a Chinese cohort, patients who experienced a COVID-19-related delay in surgical treatment had more aggressive cancer behavior. Patients treated after lockdown were more likely to have multiple lesions (31.2% vs 36.5%, *p* = 0.040), extrathyroidal extension (65.5% vs 72.2%, *p* = 0.011), and lymph node metastases (37.7% vs 45.0%, *p* = 0.007) [17].

In our experience [18], delayed treatment did not increase the rate of extrathyroidal extension, lymph node, and distant metastases, extracapsular extension, or incomplete surgical resection. However, it slightly increased the rate of high-risk patients (19.4% vs 5.5%; *p* = 0.036), according to the ATA risk stratification. In any case, the response to treatment 1 year after the initial treatment was not affected by the delay, which actually led to a better risk-stratification and risk-benefit balance. All patients underwent a risk-stratification triage, which prompted to active surveillance programs in those cases harboring low-risk papillary microcarcinomas, as already suggested by recent evidence and clinical practice guidelines [19].

## The patients’ views

Even if a delay in treatment of newly discovered thyroid cancers that pose no clinical risks is completely acceptable by physicians who are aware of the recent evidence and recommendations, the patients’ experiences may be negative, as the perceived risk may be overestimated.

During the COVID-19 pandemic, thyroid cancer survivors reported increased anxiety compared to a pre-COVID cohort [20]. In our thyroid cancer patients, who reported substantial emotional distress, its degree was similar across the entire broad range of disease statuses. In the multivariate analysis, disease severity and stage were not significant predictors of the degree of distress. These findings suggest that “cancer patients” are experiencing substantial adverse psychological effects from the SARS-CoV2 outbreak that are largely unrelated to their objective healthcare needs [21].

A similar UK survey identified a negative impact of COVID-19 outbreak and lockdowns on psychological wellbeing of thyroid patients (including patients with benign diseases such as primary hypothyroidism and hyperthyroidism). These patients reported low overall satisfaction rates, due to cancellations of appointments and planned treatments [22].

To deliver comprehensive care, clinicians should listen and understand patient concerns and improve their communication about risk-benefit balances of different treatment plans.

The COVID-19 pandemic might therefore have started a virtuous process and be an opportunity to reduce the unnecessary diagnostic and therapeutic burden for thyroid cancer patients. However, there is still a way to go toward full implementation and acceptance of recommendations within the wider endocrine and surgical communities to reduce the unnecessary interventions. Furthermore, this requires better physician–patient communication and truly shared decision-making process.
